# Safety and Tolerance of a Novel MMR (Measles, Mumps, and Rubella) Vaccine in Indian Children: A Real-World Evidence Study

**DOI:** 10.7759/cureus.100089

**Published:** 2025-12-25

**Authors:** Jagannatha R Pula, Pradeep K, Rakeshkumar M Patel, Sanjay V Mankar, Sapan Vinayak, Santanu Ray, Sabyasachi Bhattacharjee, Piyush Jain, Poonam Bhatia, Trayambak Dutta, Manish Mahajan

**Affiliations:** 1 Pediatrics, PJR Children's Clinic, Bengaluru, IND; 2 Pediatrics, Little Star Child Care and Vaccination, Hyderabad, IND; 3 Pediatrics, Amruta Hospital, Rajkot, IND; 4 Pediatrics, Mankar Hospital, Pune, IND; 5 Pediatrics, Private Practice, New Delhi, IND; 6 Pediatrics, Protyasa Clinic New Alipore, Kolkata, IND; 7 Pediatrics, M N Gupta Health Clinic, Kolkata, IDN; 8 Pediatrics, Private Practice, New Delhi, IND; 9 Infectious Disease, Zydus Lifesciences Limited, Ahmedabad, IND; 10 Medical Affairs, Zydus Lifesciences Limited, Ahmedabad, IND

**Keywords:** immunisation, measles mumps rubella vaccine, real world evidence, safety, tolerability

## Abstract

Background

Measles, mumps, and rubella continue to cause outbreaks and serious complications in many regions. Routine childhood vaccination is the most effective strategy for preventing these infections. Multiple MMR vaccines are used in India, including both domestic and international formulations. The objective of this study was to evaluate the safety and tolerability of the ZyVac MMR vaccine (Zydus Lifesciences Ltd., Ahmedabad, Gujarat, India) administered during routine paediatric practice.

Methods

This retrospective post-marketing safety study was conducted across nine paediatric centres in India from August 2025 to November 2025. Children aged 9-26 months who received a single 0.5 mL subcutaneous dose of ZyVac MMR vaccine were included. Solicited local and systemic adverse events (AEs) were recorded on Day 0 retrospectively and on Day 7, prospectively, wherein Day 0 was the day of vaccination. Unsolicited or serious AEs were monitored through Day 7. Caregivers and clinicians independently rated vaccine tolerability using a standard global assessment scale. Descriptive statistics were used to summarise AE incidence.

Results

A total of 381 children were vaccinated. No serious AEs were reported. Overall, 3.67% of children experienced AEs. Fever (2.1%) and rash (0.79%) were the most common reactions. Local AEs, including pain and redness, occurred in 0.26% each. One child reported mild rhinorrhoea. All events were mild, brief, and resolved without treatment. More than 99% of caregivers and investigators rated tolerability as “excellent” or “good.”

Conclusion

ZyVac MMR demonstrated an excellent safety and tolerability profile in Indian children during routine clinical use, with only mild, self-limiting AEs observed. These findings support its inclusion in early childhood immunisation schedules and reinforce current recommendations in India for timely MMR administration to strengthen population-level immunity.

## Introduction

Measles, mumps, and rubella (MMR) continue to pose substantial global public health challenges due to their high transmissibility and potential for severe complications. Measles remains one of the most contagious viral infections, with an estimated 10.3 million cases reported worldwide in 2023 [1]. In the absence of specific antiviral therapy, clinical management is supportive and includes vitamin A supplementation and treatment of complications such as pneumonia, diarrhoea, and encephalitis [2]. Mumps infection typically presents with parotitis but may progress to orchitis, pancreatitis, aseptic meningitis, encephalitis, or permanent sensorineural hearing loss. Treatment is largely symptomatic [3]. Rubella is generally a mild illness in children; however, infection during pregnancy carries a high risk of miscarriage, stillbirth, or congenital rubella syndrome (CRS), which is associated with congenital heart defects, cataracts, hearing impairment, and neurodevelopmental delay. In 2019, an estimated 32,000 infants globally were affected by CRS, reflecting persistent immunity gaps [4].

India has made significant progress in reducing the incidence of measles and rubella through the Universal Immunisation Programme (UIP) and the nationwide measles-rubella (MR) vaccination campaign. By early 2025, MR first-dose and second-dose coverage had reached approximately 93.7% and 92.2%, respectively, contributing to marked reductions in measles and rubella cases between 2023 and 2024 [5]. Periodic mumps outbreaks reported across several states further indicate continued susceptibility and emphasise the importance of broad MMR immunisation. The Indian Academy of Pediatrics (IAP) Advisory Committee on Vaccines and Immunization Practices (ACVIP) recommends at least two doses of the MMR vaccine, a month apart, as catch-up for those vaccinated with the Government of India’s MR vaccine under UIP. Intermittent outbreaks and ongoing CRS cases highlight the need for sustained high vaccination coverage, timely administration of the first dose, and strengthened surveillance systems [6].

Several licensed MMR vaccines are available in India, most commonly formulated with the Edmonston-Zagreb measles strain, the Leningrad-Zagreb mumps strain, and the RA 27/3 rubella strain. The Leningrad-Zagreb mumps strain has been associated with higher reported rates of aseptic meningitis compared with alternative mumps strains [7]. These observations have stimulated interest in MMR vaccine formulations incorporating mumps strains with more favourable safety profiles.

ZyVac® MMR (Zydus Lifesciences Ltd., Ahmedabad, Gujarat, India) is a live, attenuated trivalent vaccine containing the Edmonston-Zagreb measles strain, the Hoshino mumps strain, and the RA 27/3 rubella strain. According to the manufacturer’s summary of product characteristics (SmPC), ZyVac MMR received initial Indian marketing authorisation on June 30, 2016, and a multi-dose (10-dose) presentation was subsequently approved in June 2024 [8]. The Hoshino mumps strain has been used internationally for more than 25 years and is recognised for its favourable safety profile. It also features in the WHO’s technical series and the WHO position paper on mumps vaccines [9]. Pre-licensure Phase II and III clinical studies in India demonstrated acceptable immunogenicity and safety in children vaccinated at 15-18 months of age and older, and its non-inferiority with the MMR vaccine by Serum Institute of India Private Limited (Pune, Maharashtra, India) [10,11].

Although controlled clinical studies have demonstrated the safety and immunogenicity of ZyVac MMR, there is a paucity of real-world evidence, particularly for administration from nine months of age and onwards. To address this evidence gap, the present study evaluated the real-world safety of ZyVac MMR in children vaccinated from nine months of age and onwards under routine clinical conditions.

## Materials and methods

Study design and setting

This retrospective, multicentric, real-world evaluation was conducted across nine paediatric centres in India in the cities of Bengaluru, Hyderabad, Rajkot, Pune, New Delhi, and Kolkata, between August 2025 and November 2025. The study was based entirely on routinely collected clinical information generated during standard immunization practices, without introducing any modification to existing clinical workflows. All relevant data extracted from physical medical records were transcribed into a standardized electronic data capture (EDC) system to ensure consistent documentation and facilitate structured analysis.

Study population

The study included children who were due to receive the first dose of the ZyVac MMR vaccine. The IAP ACVIP recommends the first dose of the MMR vaccine at nine months of age. A consecutive sampling method was followed to include all eligible cases with complete vaccination and Day 7 follow-up data. Children were required to be clinically stable at the time of vaccination. Exclusion criteria, as documented in the medical records, included any prior measles, mumps or rubella infection or MMR vaccination, history of severe allergic reaction to vaccines, recent exposure to measles or rubella, major neurological or chronic systemic illness, immunosuppression, acute febrile illness at the time of vaccination, recent receipt of blood products or other vaccines, and documented contact with a confirmed measles or rubella case.

Study objectives

The primary objective was to assess the safety and tolerability of the ZyVac MMR vaccine under routine clinical conditions. Safety assessments included solicited local and systemic adverse events (AEs) recorded on Day 0 and Day 7, as well as unsolicited and serious AEs occurring within seven days following vaccination. Tolerability was independently assessed by caregivers and investigators using the FACT-GP5. This was used under the Functional Assessment of Chronic Illness Therapy (FACIT) Licensing Agreement.

Intervention

All enrolled children had received a single 0.5-mL subcutaneous dose of the ZyVac MMR vaccine administered into the anterolateral thigh, as per standard immunization practices and IAP ACVIP recommendations. The study did not influence the vaccination decision or procedure, and no additional interventions or monitoring were undertaken beyond usual clinical care.

Study procedures and follow-up

Day 0 clinical records included demographic characteristics, medical history, concomitant medications, vaccination details, and documentation of baseline solicited local reactions. Day 7 follow-up data captured solicited systemic AEs along with any unsolicited events recorded during the seven-day interval. For every AE, information on onset, duration, severity, clinical description, treatment provided, and outcome was extracted directly from the source records. Caregivers and investigators independently completed the Global Assessment of Tolerability scale during the follow-up visit.

Outcome measures

The primary outcomes were the incidence and profile of solicited local and systemic AEs on Day 0 and Day 7, and the occurrence of unsolicited and serious AEs within seven days of vaccination. Secondary outcomes included overall tolerability assessments completed by caregivers and investigators. Causality assessment was performed using the WHO-Uppsala Monitoring Centre (UMC) system to classify AE-vaccine relationships.

Statistical analysis

All extracted clinical data were entered into the EDC system for analysis. Records with incomplete essential information were excluded, and no data imputation was performed. Continuous variables were summarized using means and standard deviations or medians and interquartile ranges based on distribution properties. Categorical variables were described using frequencies and percentages. Comparative analyses of Day 0 and Day 7 findings were conducted using appropriate parametric or non-parametric tests, and statistical significance was defined as p < 0.05.

Ethical considerations

The study was approved by the ACEAS-Independent Ethics Committee, Ahmedabad (Protocol No. ST/ESMO0425/08). Only de-identified retrospective clinical data were used for analysis, ensuring protection of patient confidentiality. All procedures complied with national regulatory requirements, Good Clinical Practice guidelines, and the ethical principles of the Indian Council of Medical Research.

## Results

Demographic characteristics

**Figure 1:**
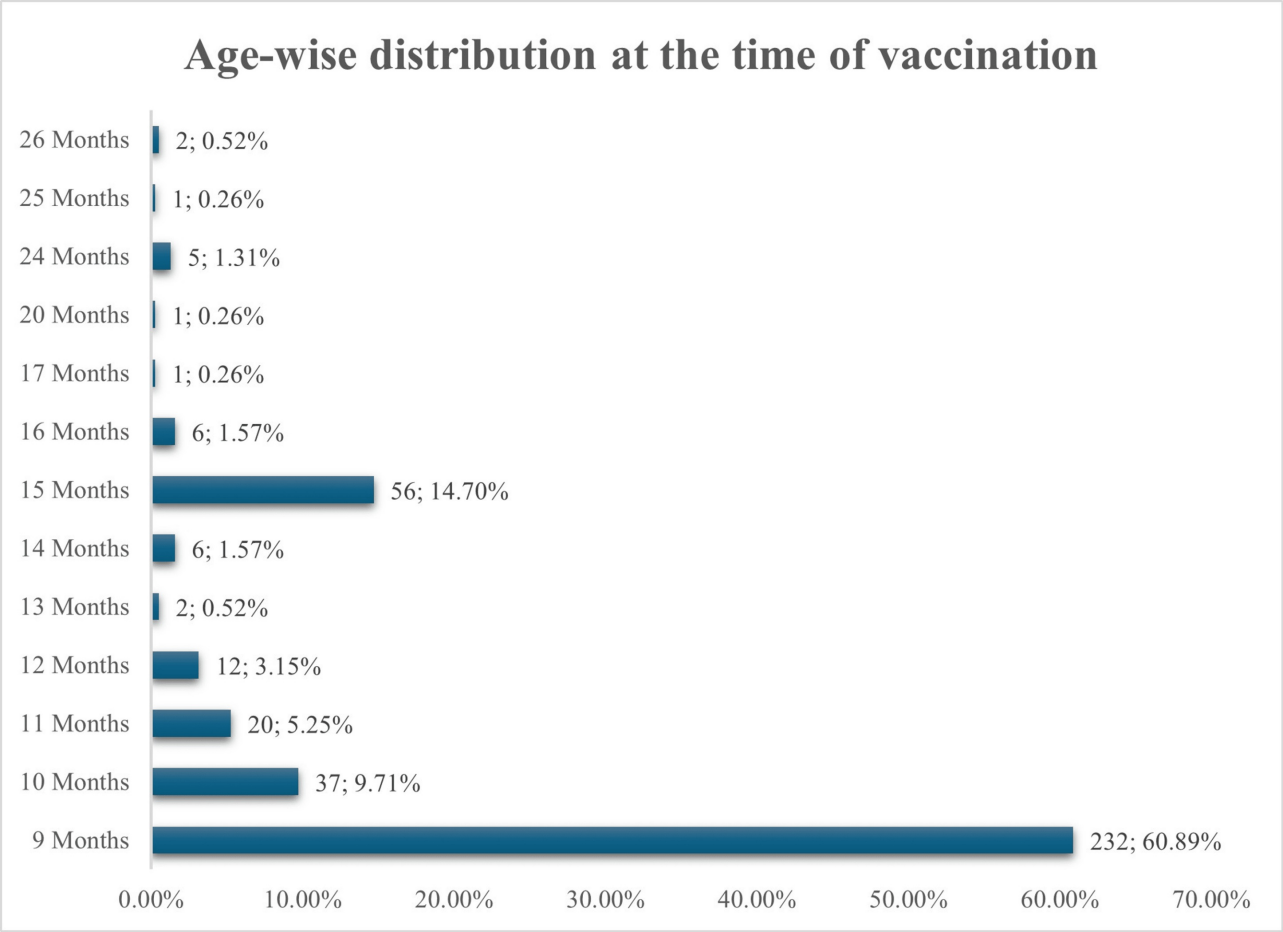
Age-wise distribution of study participants at the time of ZyVac* MMR vaccination (N=381) Data presented as n; % * Zydus Lifesciences Limited, Ahmedabad, Gujarat, India MMR: Measles, Mumps, and Rubella

The study enrolled 381 participants who received the ZyVac MMR vaccine. The median age at vaccination was nine months (interquartile range (IQR): 9-11 months), with the majority (60.89%, n = 232) vaccinated precisely at nine months. Overall, 75.85% (n = 289) were vaccinated between nine and 11 months of age. Delayed vaccination was observed in a smaller proportion: 14.70% (n = 56) at 15 months and 9.45% (n = 36) between 12 and 26 months (Figure 1).

The mean anthropometric measurements were consistent with expected norms for this age group: mean weight 8.89 kg (SD ± 1.72 kg) and mean height 73.73 cm (SD ± 12.03 cm). The cohort was predominantly male (64.3%, n = 245), with female infants comprising 35.7% (n = 136), resulting in a male-to-female ratio of approximately 1.8:1 (Table 1).

**Table 1:** Sex-wise distribution of study participants (N=381)

Sex	Frequency (Percentage)
Female	136 (35.7%)
Male	245 (64.3%)

Safety profile

**Figure 2:**
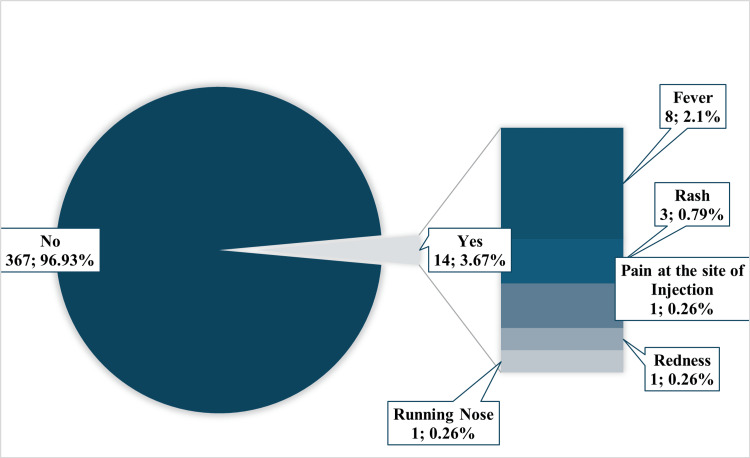
Distribution of study participants according to the nature of reported AEFIs (N=381) Data presented as n; % AEFI: adverse events following immunization

The ZyVac MMR vaccine showed a strong safety profile in this real-world study. Only 3.67% (n=14) of vaccinated individuals reported AEs following immunization (AEFIs) (Table 2). The most common AEFI was fever, occurring in 2.1% (n=8) of participants, followed by rash in 0.79% (n=3). Local reactions at the injection site were infrequent, with pain and redness each reported in 0.26% (n=1) of cases, and one participant reported a runny nose in 0.26% (n=1) (Figure 2). Notably, all AEFIs were solicited, classified as mild, and resolved spontaneously on the same day without requiring any medical management.

**Table 2:** Distribution of study participants according to reported AEFIs (N=381) AEFI: adverse events following immunization

Reported AEFIs	Frequency (Percentage)
Yes	14 (3.67%)
No	367 (96.93%)

The analysis of AEFI incidence across different geographical zones revealed a relatively uniform distribution pattern (Table 3). The South zone reported the highest frequency of AEFIs with five cases (1.31%), followed by the West zone with four cases (1.05%), the East zone with three cases (0.79%), and the North zone with two cases (0.52%). Despite these differences, the overall AEFI rates across all zones remained consistently low, ranging from 0.52% to 1.31%, suggesting comparable safety profiles independent of geographical location.

**Table 3:** Zone-wise distribution of reported AEFIs in India (N=14) Percentage count has been calculated from the total study participants AEFI: adverse events following immunization

Zone	Frequency (Percentage)
North	2 (0.52%)
West	4 (1.05%)
South	5 (1.31%)
East	3 (0.79%)
Total	14 (3.67%)

Fever, a well-known and expected post-vaccination immune response common to all MMR vaccines globally, accounted for 57.1% (8/14) of events, distributed across all zones without any clustering, indicating no region-specific concern. Rash was infrequent (21.4%, 3/14) and seen as isolated cases across three zones. Local reactions were rare, with only one case each of pain and redness (7.1% each), reflecting excellent local tolerability. A single mild respiratory symptom (running nose, 7.1%) was reported. Importantly, AEFI occurrence remained low across all age groups, with reports being minimal in children vaccinated at nine months (1.84%), 10-14 months (1.05%), and 15 months and above, indicating a consistently favourable safety profile across the recommended age spectrum.

Overall, the findings reinforce that ZyVac MMR demonstrates a strong and predictable safety profile, consistent with established global evidence for MMR vaccines and without any signals of concern across regions or age groups. AEFI occurrence was low across all age groups, children vaccinated at nine months (1.84%), followed by those vaccinated at 10-14 months (1.05%), 15 months (0.52%), and >15 months (0.26%) (Table 3). 

No unsolicited AEs or serious AEs were reported in this study. Zone-wise distribution by age showed no clustering pattern; AEFIs in the nine-month group were reported from all four zones, whereas events in older age groups were distributed across one or two zones without any regional predominance (Table 4). Overall, AEFI patterns remained uniformly low and scattered across ages and regions.

**Table 4:** Zone-wise distribution of reported AEFIs in study participants stratified by age at vaccination (N=14) Percentage count has been calculated from the total study participants AEFI: adverse events following immunization

Zone	9 months at vaccination, n (%)	10-14 months at vaccination, n (%)	15 months at vaccination, n (%)	>15 months at vaccination, n (%)	Total, n (%)
North	1 (0.26%)	1 (0.26%)	0	0	2 (0.52%)
West	2 (0.52%)	1 (0.26%)	1 (0.26%)	0	4 (1.05%)
South	3 (0.79%)	1 (0.26%)	1 (0.26%)	0	5 (1.31%)
East	1 (0.26%)	1 (0.26%)	0	1 (0.26%)	3 (0.79%)
Total	7 (1.84%)	4 (1.05%)	2 (0.52%)	1 (0.26%)	14 (3.67%)

Tolerability profile

Patient-Reported Tolerability

**Figure 3:**
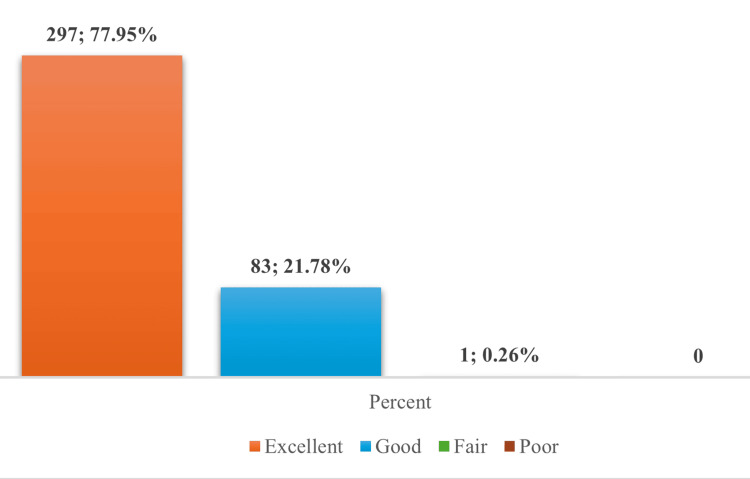
Participant-reported tolerability outcomes following ZyVac MMR vaccination (N=381) Data presented as n; % MMR: Measles, Mumps, and Rubella

Patient-reported tolerability assessments revealed overwhelmingly positive outcomes, with 77.95% (n=297) rating tolerability as "excellent" and 21.78% (n=83) as "good." Only one participant (0.26%) reported "fair" tolerability (Figure 3).

Physician-Assessed Tolerability

**Figure 4:**
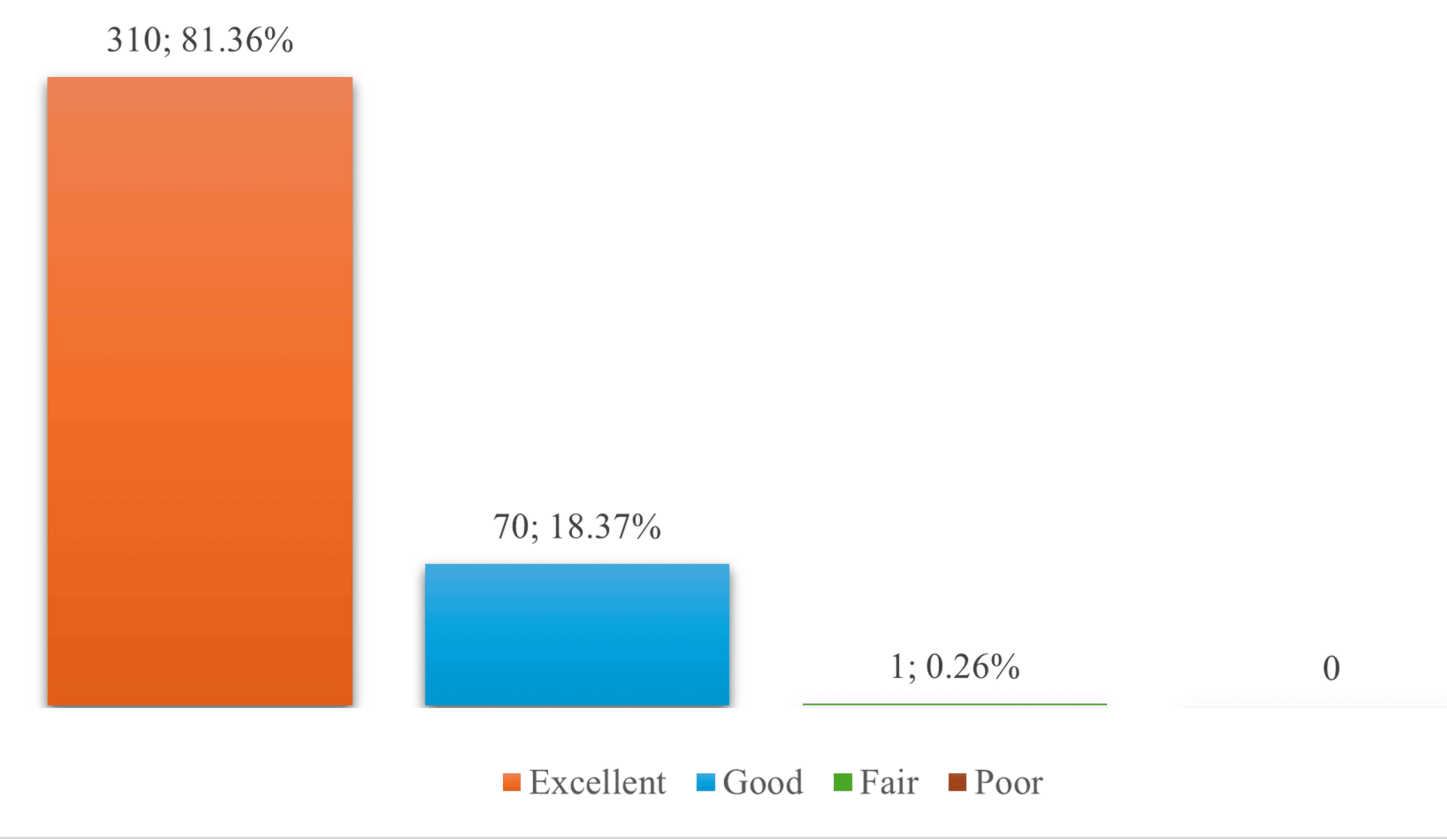
Physician-reported tolerability outcomes following ZyVac MMR vaccination Data presented as n; % MMR: Measles, Mumps, and Rubella

Physician assessments corroborated patient reports, with 81.36% (n=310) of healthcare providers rating vaccine tolerability as "excellent" and 18.37% (n=70) as "good." Similar to patient reports, only one case (0.26%) was rated as "fair” (Figure 4).

## Discussion

This post-marketing surveillance assessed the safety and tolerability of the ZyVac MMR vaccine administered during routine paediatric immunisation sessions across nine centres in India. The study population consisted of infants of routine paediatric practice as guided by IAP ACVIP [6]. All children were followed through standard clinical workflows, and AEs were captured retrospectively. No serious AEs were reported, and the incidence of mild, self-limiting events remained low.

The observed AE rate of 3.67% is lower than rates typically reported in controlled clinical trials for MMR vaccines. Previous Phase II data showed fever in 15.4% of vaccine recipients and rash or rhinorrhoea in 4.1% [10], while the Phase III trial documented a 3.2% AE rate in ZyVac MMR recipients compared to 8.9% in the comparator group [11], This study complements the earlier trials by providing real-world evidence of favourable tolerability when ZyVac MMR is administered [12]. These findings support the IAP ACVIP guidelines, which emphasise timely first-dose coverage to enhance measles, mumps, and rubella control at nine months of age. Demonstrating safety at this earlier age reinforces confidence in IAP ACVIP's vaccination schedule and may help reduce immunisation delays [6].

The low frequency and mild nature of reported events align with global post-marketing data. The Hoshino mumps strain used in ZyVac has shown a strong safety record internationally, with a lower incidence of aseptic meningitis compared to other mumps strains [7,9]. Broad surveillance confirms that MMR vaccines most often cause minor reactions such as fever or rash, while serious AEs remain uncommon [13]. Maintaining high early coverage is essential for MMR disease elimination. Measles control requires approximately 95% immunity, mumps seroprotection requires two doses of the MMR vaccine, and sustained MMR or MR vaccine uptake remains central to rubella and congenital rubella syndrome prevention [14]. This study reinforces the operational feasibility and clinical safety of achieving early protection through the ZyVac MMR vaccine.

Although economic outcomes were not measured directly, the favourable safety profile and minimal disruption associated with vaccination suggest programmatic benefits. Large-scale immunisation efforts have been shown to reduce health service utilisation, caregiver absenteeism, and societal costs [15]. Prior studies have estimated strong return on investment for routine vaccination, particularly when factoring in productivity gains and hospitalisation avoidance [16]. This study's strengths include a multicentre design, representation from diverse regions, and concordant tolerability ratings from both caregivers and healthcare providers. Nonetheless, several limitations should be acknowledged. The retrospective methodology may underestimate minor AEs and cannot detect late-onset events beyond the seven-day window. Immunogenicity was not assessed, and no comparator group was included. Future studies should address these limitations by incorporating demographic and productivity data, extending follow-up durations, applying active surveillance methods, and evaluating administrative techniques. Comparative assessments with alternative MMR formulations may also offer deeper insights into real-world vaccine performance [17, 18].

Despite these limitations, the present study offers valuable real-world evidence supporting early administration of the ZyVac MMR vaccine and contributes to the growing body of literature affirming the safety of mumps strains with favourable profiles such as Hoshino [9]. Future research should prospectively collect safety and immunogenicity data from a larger, more diverse cohort, extend follow-up beyond seven days, and include comparator arms. Extended observations can also capture productivity and economic outcomes, which have been shown in other settings to yield substantial cost savings and societal benefits when vaccines reduce disease burden [16]. Maintaining high coverage at or above the herd-immunity threshold (around 95% for measles) is essential to protect communities and prevent outbreaks and these findings underscore the feasibility of achieving this through routine immunisation in India.

## Conclusions

This real-world evaluation demonstrates that the novel MMR vaccine has an excellent safety and tolerability profile in Indian children. The vaccine was well tolerated, with no serious AEs observed and only mild, transient reactions such as fever and rash reported; this favourable safety and acceptability profile indicates that it can be confidently integrated into routine paediatric practice, aligning with prior clinical trial findings. From a public health perspective, the availability of a safe, well-tolerated MMR vaccine supports national immunisation efforts as India strives to eliminate measles and rubella. Early MMR immunisation (initiating at ≥9 months of age) is particularly relevant for closing immunity gaps and attaining the population-level immunity needed to prevent outbreaks. Overall, these findings provide important evidence to guide clinicians and policymakers, underscoring that widespread early use of this novel MMR vaccine will help protect children and advance national goals in MMR control.
